# News and Events of the Week

**Published:** 1911-05-06

**Authors:** 


### News and Events of the Week.

The King of Italy will be present at the inaugural meeting of the Seventh International Congress on Tuberculosis, which takes place at Rome on September 24 next, Professor Baccelli being the president and Dr. Ascoli general secretary.

We have received a copy of a new monthly journal, "L'Echo Médical et Pharmaceutique," which proposes to review the scientific and professional interests of medicine and pharmacy. It is published at 50c. a number, or post free 6 francs a year in France, 8 francs a year abroad.

The foundation "Adolph von Rath" which owes its existence to the generosity of Madame von Rath and has as its object the gratuitous distribution of nourishment to the tuberculous poor of Berlin, was responsible during the past year for 125 patients. In all, up to the present time, it has distributed 45,445 meals to 1,001 patients. This charity was founded with an endowment of half a million marks, or roughly £25,000.

After many years of varying fortune, the foundation of a medico-legal institute worthy of Paris would seem likely to be accomplished. An agreement has been come to between the State and the city by which the cost is to be divided between the two. The new institute will be erected in the place Mazas (Pont d'Austerlitz), and will comprise three distinct sets of offices. One will be given over to the administration, the dead-houses, etc., installed in quarters infinitely more suitable than the doubtful ones now occupied by the morgue. A second will provide accommodation for post-mortem examinations and judicial operations, and will consist of three autopsy rooms, with a laboratory for medico-legal examinations. A third will provide a lecture theatre with 200 places for medico-legal teaching, a laboratory for toxicology, accommodation for animals used for experimental purposes, a library and museum.

At the annual general meeting of Dr. Barnardo's Homes the Duke of Somerset was re-elected president; Mr. Howard Williams, treasurer; and the vice-presidents, the Council, and auditors were appointed for the ensuing year. The forty-fifth annual report was presented, which showed that to December 31 last, 73,251 children had been dealt with. In 1910, 2,815 children were admitted (2,243 permanently and 572 temporarily); 977 young emigrants were sent out during the year (962 to Canada and 15 to Australia and New Zealand), making a total of 22,614. Ninety-eight per cent. are successful. The death rate covering all of the Homes was remarkably low—only 6.28 per 1,000, and this notwithstanding that of this great family 1,065 were babies under five years of age and 944 belonged to the sick, ailing, blind, deaf and dumb, and incurable class. The total income for the year was £232,474. The King and Queen became patrons of the Homes during the year, Queen Alexandra already being a patron. H.R.H. Princess Henry of Battenburg became patron of her Majesty's Hospital, a branch of the Homes. Several Royal visits were paid to the Homes during the year. Two principal extensions were in progress—the Boys' Garden City, at which foundation-stones were laid in 1910 by Prince and Princess Alexander of Teck, and the erection of a much-needed hospital at the Girls' Village Home, for which funds have been raised in Australia and New Zealand, and the foundation-stone of which was laid last year by the High Commissioner for Australia.

Dr. John Attfield, F.R.S., of Watford, Herts, at one time Demonstrator of Chemistry at St. Bartholomew's Hospital, who died on March 18 last, aged seventy-six years, left estate valued at £41,275 gross and £22,163 net.

The King has been pleased to give directions for the appointment of Mr. Louis Gabriel Barbeau, M.B., Acting Director of the Medical and Health Department, and Mr. Hassan Sakir, M.B., to be Nominated Members of the Council of Government of the Colony of Mauritius.

A portrait of Professor John Cleland, who from 1877 to 1909 occupied the chair of Anatomy at Glasgow University, has been presented to the University, and another portrait of him to Mrs. Cleland. Before the presentations, the Senate met and conferred on Professor Cleland the honorary degree of LL.D.

An order came into force last Monday making it compulsory upon medical officers of hospitals to notify all cases of pulmonary tuberculosis among out-patients or in-patients. The notification is to be made to the medical officer of health for the area in which the hospital is situated within forty-eight hours after the first recognition of the disease.

The Drapers' Company has granted £6,000 to the Battersea Polytechnic for the erection and equipment of a department of hygiene and physiology. The new buildings will include a lecture theatre, class rooms, and separate laboratories for bacteriology, physiology, geology, and hygiene, with suitable rooms for the preparation of experiments.

Mr. Ivor Back, F.R.C.S., Assistant-Surgeon to St. George's Hospital, and Surgeon to Out-patients, Royal Waterloo Hospital, and Professor David Hutchison Macgregor, M.A. (Edinburgh and Cambridge), Professor of Economics in the University of Leeds, have been elected to A.K. Travelling Fellowships at the University of London.

The committee of the proposed dispensary at Battersea for the treatment and prevention of consumption has decided to secure premises in Albert Bridge Road, Battersea Park, and to appoint a medical officer and nurse, who will devote the whole of their time to the work. It is hoped that the institution will be ready for work by June 1. The expenditure for the first year is expected to be about £1,000, towards which less than £100 has at present been received.

We have received a copy of the Social Guide for 1911, edited by Mrs. Hugh Adams and Miss Edith A. Browne, and published by A. and C. Black at a price of 2s. 6d. net in cloth or 3s. 6d. net in full leather. The book gives an accurate and fairly full account of all the chief attractions from a social point of view which are to take place during the present season. As might be expected, the Coronation holds a very prominent position in the book, and a full account is given of the procession, the ceremony in the Abbey, the dress to be worn by the various ranks of society, and the celebrations in honour of the event. The book should be particularly useful to visitors to London during the year who want a handy account of "what is going on."

